# Evaluation of the frequency and predictive factors of cannabis consumption among students of the university of pharmacy of monastir

**DOI:** 10.1192/j.eurpsy.2021.1522

**Published:** 2021-08-13

**Authors:** N. Bensaid, A. Ghorbel, K. Ben Hmida, S. Hachicha, S. Smaoui, F. Messadi, J. Aloulou

**Affiliations:** 1 Toxicology Unit, hygiene Loboratory, sfax, Tunisia; 2 Psychiatrie, Hopital Hedi Chaker, sfax, Tunisia; 3 Microbiology Unit, hygiene Loboratory, sfax, Tunisia; 4 Microbiology, hygiene laboratory, sfax, Tunisia

**Keywords:** Cannabis, students, Risk factors, dependence

## Abstract

**Introduction:**

In Tunisia, the use of cannabis has become an important phenomenon in the society that not well attested by epidemiological studies.

**Objectives:**

The purpose of this work is to study the socio-demographic criteria of student of the faculty of Pharmacy of Monastir and risk factors encouraging them to consume cannabis and to estimate the extent of the phenomenon of dependence occurring among students.

**Methods:**

A questionnaire composed of 45 questions was sent to students of the Faculty of Pharmacy of Monastir via the internet. The data was collected and analyzed using the software’ Google forms’ and ‘statistical package for social Science’ (SPSS)

**Results:**

The prevalence of cannabis use among students of the faculty of Pharmacy was worrying, increasing to 21.4 %, for moments of pleasure and relaxation of the stressful life (p=0 et p=0.008). The frequency of cannabis use had a significant impact on the desire to stop cannabis use (p=0.012). Of the 55 consumers, only 5 tried to stop or reduce cannabis use. About 88% of participants indicated that cannabis was responsible for psychological dependence. Polydrug use (tobacco, alcohol, other drugs) had a significant influence on this use (p=0).

**Conclusions:**

The scourge of cannabis use has invaded not only universities, but also high schools and colleges. At the end of this work, we propose debates to find the necessary means to protect cannabis users cannabis and treat dependents.

